# The differentiation state of small intestinal organoid models influences prediction of drug-induced toxicity

**DOI:** 10.3389/fcell.2025.1508820

**Published:** 2025-01-23

**Authors:** Jessica A. Klein, Julia D. Heidmann, Tomomi Kiyota, Aaron Fullerton, Kimberly A. Homan, Julia Y. Co

**Affiliations:** ^1^ Complex In Vitro Systems, Translational Safety, Genentech Inc., South San Francisco, CA, United States; ^2^ Investigative Toxicology, Translational Safety, Genentech Inc., South San Francisco, CA, United States

**Keywords:** gastrointestinal toxicity, intestinal toxicity, diarrhea, organoids, small intestine, tissue-derived stem cells

## Abstract

Drug-induced intestinal toxicity (GIT) is a frequent dose-limiting adverse event that can impact patient compliance and treatment outcomes. *In vivo,* there are proliferative and differentiated cell types critical to maintaining intestinal homeostasis. Traditional *in vitro* models using transformed cell lines do not capture this cellular complexity, and often fail to predict intestinal toxicity. Primary tissue-derived intestinal organoids, on the other hand, are a scalable Complex *in vitro* Model (CIVM) that recapitulates major intestinal cell lineages and function. Intestinal organoid toxicity assays have been shown to correlate with clinical incidence of drug-induced diarrhea, however existing studies do not consider how differentiation state of the organoids impacts assay readouts and predictivity. We employed distinct proliferative and differentiated organoid models of the small intestine to assess whether differentiation state alone can alter toxicity responses to small molecule compounds in cell viability assays. In doing so, we identified several examples of small molecules which elicit differential toxicity in proliferative and differentiated organoid models. This proof of concept highlights the need to consider which cell types are present in CIVMs, their differentiation state, and how this alters interpretation of toxicity assays.

## 1 Introduction

Gastrointestinal toxicity (GIT) causes adverse events (AE) that can limit patient compliance, impact dosing regimens, and thus reduce treatment efficacy for therapeutics. Unfortunately, AEs such as diarrhea, nausea and vomiting are challenging to predict in preclinical studies. Animal-human translation is relatively poor for the gastrointestinal system (Olson et al., 2000; Monticello et al., 2017; Clark and Steger-Hartmann, 2018), and until recently, standard human *in vitro* models could not capture representative cell populations of the gut. Consequently, GIT often remains undetected or overlooked until clinical trials, at which point symptom management becomes the primary resolution (Cook et al., 2014; Al-Saffar et al., 2015; Hwang et al., 2016). Focusing efforts to improve human preclinical *in vitro* models that identify GI risk could mitigate this burden to patients and the drug development cycle (Al-Saffar et al., 2015; Peters et al., 2020). Because safety predictions are subject to properties of the model system, it is crucial to consider the cell types present in a model to improve *in vitro-in vivo* translation.

The small intestine epithelium is comprised of repeating units of proliferative zones (crypts) with stem and progenitor cells that differentiate to form intestinal protrusions (villi) harboring the major cell types of the intestine. Work by Sato *et al* demonstrated that by harvesting and embedding the crypts of intestinal tissue in an extracellular matrix supplied with growth factors, intestinal stem cells form 3D structures, termed organoids, with the capacity to differentiate into major cell types of the intestine (Sato et al., 2009). Intestinal tissue-derived organoids can be propagated prior to differentiation and are amenable to high throughput screening. Furthermore, they have shown significant improvement over traditional transformed cell lines, such as Caco-2, in recapitulating intestinal physiology. This includes expression of key cellular differentiation markers, mucus secretion, barrier integrity, and drug metabolism and disposition (Zachos et al., 2016; Yamashita et al., 2021; Takahashi et al., 2022; Kourula et al., 2023).

While primary tissue-derived complex *in vitro* models (CIVMs) are at the early stages of evaluation for use in drug development, they demonstrate considerable potential in predicting clinical diarrhea from small molecules (Peters et al., 2019; Belair et al., 2020; Pike et al., 2024). Given the differences in functionality between the proliferative crypts and differentiated villi of the intestine, it’s plausible that these compartments interact differently with toxicants. For instance, actively-dividing cells in the crypts may be more susceptible to chemotherapeutic-induced toxicity (Boussios et al., 2011). A recent study conducted in primary tissue-derived proliferative and differentiated intestinal monolayers corroborated that post-mitotic cellular populations are indeed less vulnerable to toxicity from anti-proliferative small molecule oncology drugs (Pike et al., 2024). Differences in susceptibility to small molecule toxicity between proliferative and differentiated cell populations in organoids however, has yet to be examined. As a proof of concept, we developed proliferative and differentiated organoid models of the small intestine (duodenum) to demonstrate how different cell populations may influence toxicity readouts. Our results indicate that prediction of diarrhea, and potentially other types of GITs, can be subject to the cellular differentiation state of the organoid models. Therefore, care must be taken in choosing the differentiation state of an organoid model for assaying GIT, especially if the mechanism of toxicity (MoT) is unknown.

## 2 Methods

### 2.1 Intestinal organoid derivation

Organoids were derived as previously described (Co et al., 2023) from duodenal tissues procured post-mortem from deidentified donors by Donor Network West. Briefly, tissues were opened longitudinally and epithelium was scraped and minced, using razor blades, into Advanced DMEM/F12 medium (Thermo Fisher Scientific) with 0.1 mg/mL Primocin (InvivoGen). The minced epithelium was resuspended in 2.5 mM EDTA in phosphate buffered-saline without Mg^2+^ or Ca^2+^ and incubated at 37°C for 9–10 min with intermittent vortexing until crypts were released (visualized using brightfield microscopy). Crypts were collected in Advanced DMEM/F12 with Primocin and filtered through a 500 μm strainer, then resuspended in Cultrex Reduced Growth Factor Basement Membrane Matrix, Type II (BME, cat. no. 3533-01002; R&D Systems) on ice and cultured in 50 μL/well domes in a 24-well plate (Corning). The domes were cured at 37°C for at least 10 min then overlaid with 0.5 mL passage medium consisting of IntestiCult™ Human Intestinal Organoid Growth Medium (OGM, cat. no. 06010; STEMCELL Technologies) supplemented with 0.1 mg/mL Primocin, 10 μM ROCK inhibitor Y-27632 (cat. no. 72304; STEMCELL Technologies) and 2.5 μM GSK-3 inhibitor CHIR 99021 (cat. no. 4423; Tocris Bioscience). Passage medium was replaced after 2–3 days with growth medium (OGM absent ROCK and GSK-3 inhibitors) and replenished every 2–3 days thereafter.

### 2.2 Organoid maintenance and differentiation

Duodenum organoids were passaged every 1–2 weeks by dissociation in approximately 0.5 mL of TrypLE™ Express Enzyme (cat. no. 12604013; ThermoFisher) per 50 μL BME at 37°C for 10 min and trituration to single cells using a P1000 pipette. An equal volume of room temperature PBS was added to inactivate the TrypLE™ and the cellular suspension was pelleted by centrifugation for 3 min at 450 × g. The cells were resuspended to a density of approximately 6 × 10^5^ cells/mL in BME on ice and plated as 50 μL domes. The domes were cured and overlaid with passage medium that was replenished with growth medium every 2–3 days, as described above. After 7 days of culture in OGM, organoids were maintained in OGM or washed with Advanced DMEM/F12 and transitioned to differentiation medium (IntestiCult™ Human Intestinal Organoid Differentiation Medium (ODM), cat. no. 100–0214; STEMCELL Technologies) supplemented with 0.1 mg/mL Primocin.

### 2.3 Transcriptomic analysis

Organoids from Donor 1 in BME domes were cultured in Proliferative conditions (7 days in OGM growth medium) or in Differentiation conditions (7 days in OGM growth media, followed by an additional 4 days in differentiation medium) after 7 days in growth medium (n = 3 wells for each condition). Samples were collected in RLT Plus lysis buffer and stored at −80°C. RNA extraction, bulk mRNA-seq (NovaSeq PE150) and analysis was performed by Novogene. Reads were aligned using HISAT220, differential gene expression analysis was performed using DESeq221, and statistical significance was calculated using the negative binomial distribution model with Benjamini–Hochberg FDR correction. PCA analysis was performed using the gene expression value (FPKM) of all samples. The full dataset is available through NCBI GEO under accession number GSE277613.

### 2.4 Compound dose-response and cell viability assay

Organoids were grown for 7–10 days then dissociated using TrypLE™ Express Enzyme, as described above. Single cells were resuspended in BME at a density of 5–6 × 10^5^ cells/mL on ice and plated as 5 μL/well domes in clear-bottom 96-well plates (Corning). The domes were cured for 10–15 min at 37°C and overlaid with 100 μL of growth media replaced every 2–3 days.

The following compounds used in this study: afatinib (Pharmaron), aspirin (Sigma), colchicine (Jecure), nifedipine (Sigma) and sorafenib (LC Laboratories), were prepared as 20 mM (500 mM for aspirin) stock solutions in DMSO and stored at −20°C prior to use. Compounds were administered after 5–6 days in growth medium for proliferative organoids or after an additional 4 days in differentiation medium for differentiated organoids. Organoid media was replaced with 90 μL fresh media immediately prior to dosing. Compounds were diluted in growth or differentiation medium and administered as a 5-fold dilution series at 5-8 concentrations with corresponding 0.5% DMSO vehicle control (1% DMSO for aspirin), at 10 μL/well in triplicate. The final concentration dose range was from 0.00128 to 100 μM forafatinib, 0.00128 to 4 μM for colchicine, 0.032 to 100 μM for sorafenib, 0.16 to 100 μM for nifedipine, and 8 to 5,000 μM for aspirin. After 3 days of treatment, cultures were imaged by brightfield microscopy (THUNDER DMi8 inverted light microscope with a 2.5× objective and a DFC9000 GTC camera (Leica)). Wells were precluded from analysis on visual quality control (i.e., organoid loss due to BME degradation/dislodging or insufficient seeding density) congruent with cytotoxicity analysis. An equal volume of Cell Titer Glo 3D Reagent (Promega) was added to each well as described by the manufacturer’s instructions. Luminescence was measured on EnSight and EnVision (Perkin Elmer) plate readers and normalized to on-plate vehicle controls (0.5%–1% DMSO) in corresponding growth or differentiation medium. Experiments were conducted in technical triplicates and repeated in organoids from 3 different donors (donor demographics noted in Supplementary Table S1). Normalized viability was averaged at each concentration for each donor, and mean of donors was fit to a 4-Parameter Logistic (4PL) curve using Prism (GraphPad) to estimate the half-maximal inhibitory concentration (IC_50_) for each compound.

### 2.5 Statistical analysis

All statistical analyses were performed using Prism 10 software (Graphpad) unless otherwise stated. Statistical tests, n, and p-values are indicated in figure legends where applicable.

## 3 Results

### 3.1 Development and characterization of proliferative and differentiated organoid models

**FIGURE 1:**
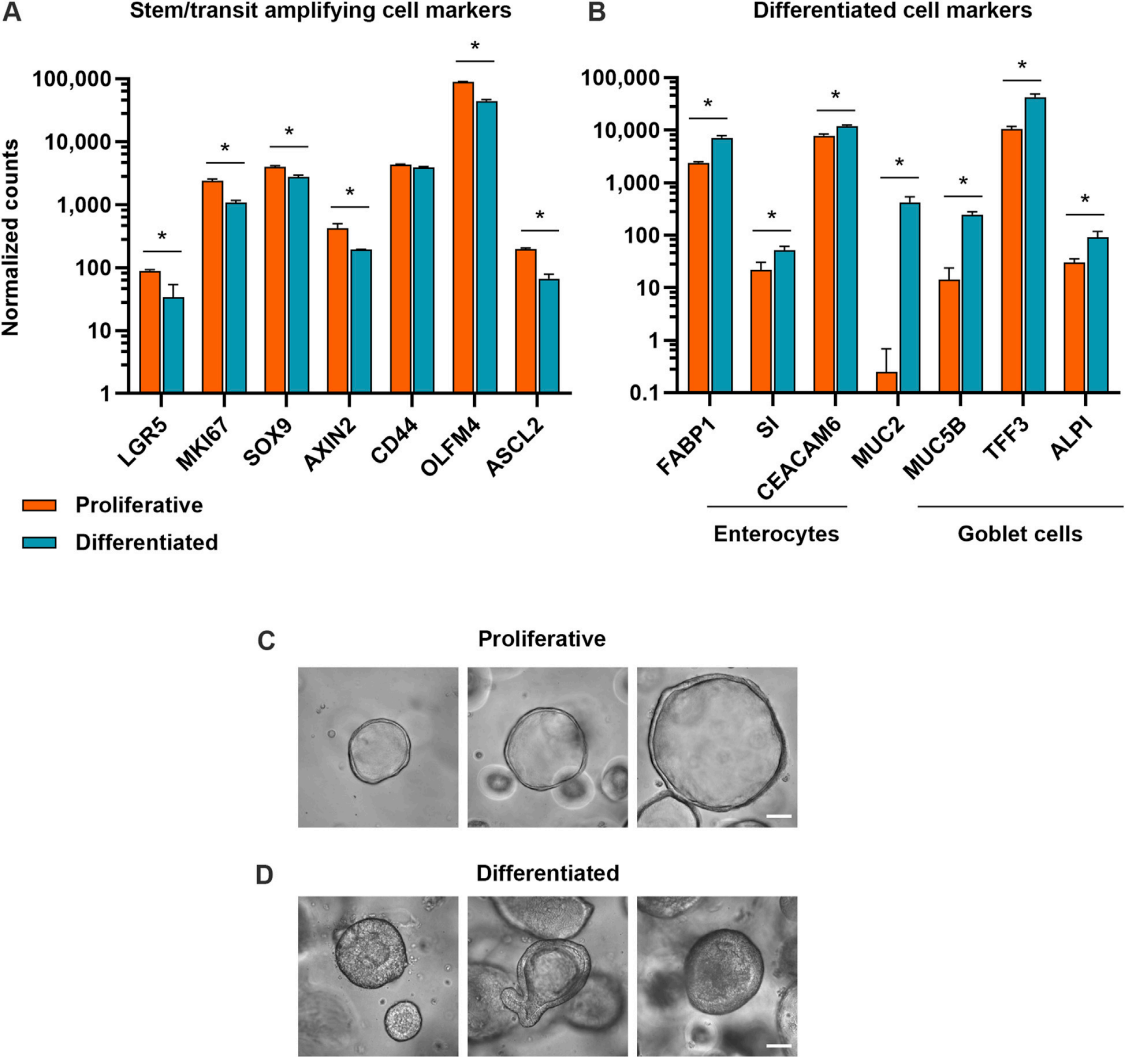
Proliferative and differentiated organoid models exhibit hallmarks of expected cell types. **(A)** Expression of stem and progenitor cell markers and **(B)** expression of enterocyte and goblet cell markers in proliferative (7 days of growth) and differentiated (7 days of growth, then 4 days of differentiation) organoid models. Data represented are mean ± SD for normalized counts determined by DESeq2, n = 3 replicates. * = p-adj <0.05. **(C)** Representative brightfield microscopy of human duodenum organoids at ×40 magnification after 5 days in growth medium and **(D)** after 5 days in growth medium, then 4 days in differentiation medium. Scale bars are 50 μm.

To investigate how proliferative and differentiated intestinal cell populations respond to various small molecule compounds, we developed a proliferative organoid model and a differentiated organoid model. Because organoids begin to spontaneously differentiate after extended duration in culture (Belair et al., 2020; Pleguezuelos-Manzano et al., 2020), proliferative organoids were cultured in IntestiCult™ Human Intestinal Organoid Growth Medium (OGM) for 5–7 days. To optimize culture conditions for the differentiated organoid model, organoids were cultured for 5–7 days before transitioning to IntestiCult™ Human Intestinal Organoid Differentiation Medium (ODM) for 4 days. Bulk RNA-sequencing verified that in the differentiation condition, expression of markers of cell proliferation and stemness (KI67, LGR5, SOX9, AXIN2, CD44, OLFM4, ASCL2) had declined relative to organoids in the proliferative condition. This coincided with an increase in expression of markers of differentiated enterocytes (FABP1, SI, CEACAM6) and goblet cells (MUC2, MUC5B, TFF3, ALPI) (Figures 1A, B). Morphologically, proliferative organoids displayed the characteristic cyst-like morphology with open lumens, while differentiated organoids had condensed lumens, taller columnar cells, budding and complex architectures (Figures 1C, D).

### 3.2 Small molecules differentially impact viability of proliferative and differentiated organoids

**FIGURE 2:**
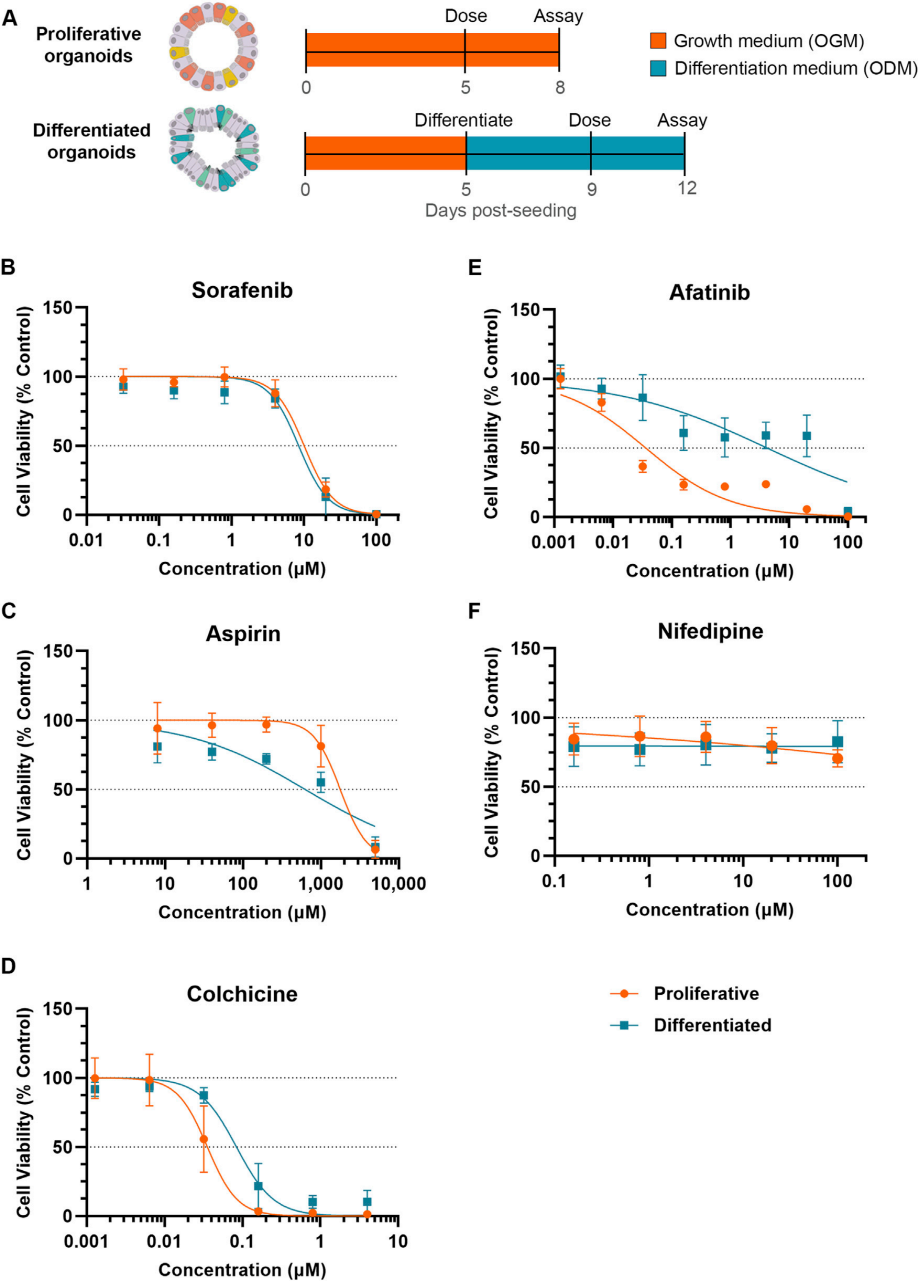
Differentiation status of organoid model can affect susceptibility to small molecule toxicity. **(A)** Schematic of experiment timeline. **(B–F)** Cell viability (Cell Titer Glo 3D) of proliferative (orange) or differentiated (blue) organoids after 3 days of exposure to small molecules normalized to respective DMSO vehicle control. Data represented as mean ± SD Small molecules tested were **(B)** sorafenib, **(C)** aspirin, **(D)** colchicine, **(E)** afatinib and **(F)** nifedipine (non-diarrheagenic example).

To compare toxicity responses of proliferative and differentiated organoids, proliferative organoids were grown 5–6 days in OGM prior to dosing, while differentiated organoids were transitioned to ODM for an additional 4 days prior to dosing (Figure 2A). Both proliferative and differentiated organoids were incubated with compounds or a 0.5%–1% DMSO vehicle control (as indicated) for 3 days prior to measuring ATP levels as a readout for cell viability. DMSO did not impact ATP levels relative to the media-only condition for either proliferative or differentiated organoids as evidenced by relative fluorescence units (Supplementary Figure S1). Compound-treated organoid ATP readouts were normalized to vehicle controls and a half-maximal inhibitory concentration (IC_50_) was calculated from dose-response curves of organoid viability for five compounds across 3 donor organoid lines.

Exposure to sorafenib, a tyrosine kinase inhibitor used to treat renal and hepatocellular carcinomas (Llovet et al., 2008) which causes diarrhea in 43% of patients (Peters et al., 2019), resulted in a decrease in organoid viability in both proliferative and differentiated organoids with IC_50_ values of 10.03 µM and 8.42 µM, respectively (Figure 2B). With similar dose response and IC_50_ values between proliferative and differentiated organoids, these data suggest that cellular differentiation state under these conditions does not influence prediction of sorafenib toxicity.

Acetylsalicylic acid (aspirin) is a non-steroidal anti-inflammatory drug (NSAID) associated with GI-related mucosal injury including ulcerations and loss of villi (Endo et al., 2015), but has a low incidence of diarrhea at less than 2% with oral dosing (Rampal et al., 2001). The MoT is understudied, but has been attributed to direct killing of enterocytes (Bjarnason et al., 1993; Endo et al., 2015). Based on this MoT we would expect that NSAIDs cause a stronger viability decrease in differentiated organoids than proliferative organoids. Decreases in viability were minimal below 100 µM in both models, however at 200 µM an approximately 25% decline in viability of the differentiated organoid model was observed, which contrasted with no decline in the proliferative organoids (Figure 2C). This resulted in an IC_50_ of 626 µM in differentiated organoids compared to 1,774 µM for proliferative organoids, suggesting that differentiated cells may be more susceptible to toxicity resulting from aspirin.

In contrast to NSAIDs which primarily affect differentiated cells, we hypothesized that compounds targeting cell cycle pathways may induce more toxicity to proliferative organoids than differentiated organoids. Colchicine is an anti-inflammatory of the alkaloid drug class primarily used to treat gouty arthritis (Slobodnick et al., 2015) with a clinical diarrhea incidence of 77% (Peters et al., 2019). *In vivo*, colchicine toxicity has been attributed to mitotic arrest through binding of tubulin, inhibiting microtubule polymerization (Daniels et al., 2008; Marginean, 2016). Because of this propensity to target actively dividing cells, we anticipated that colchicine would demonstrate more potent toxicity towards proliferative organoids. Indeed, the IC_50_ of colchicine in differentiated organoids (0.084 µM) was more than double that of proliferative organoids (0.035 µM), indicative of a higher toxicity towards the proliferative cell model (Figure 2D).

We next evaluated another antineoplastic kinase inhibitor, afatinib, which is primarily indicated for treating non-small cell lung cancer (NSCLC), and irreversibly binds human epidermal growth factor receptor 2 (HER2) and epidermal growth factor receptor (EGFR) kinases to inhibit pathways of cell growth, proliferation, and survival (Citri and Yarden, 2006; Modjtahedi et al., 2014; Keating, 2016). Afatinib is considered highly diarrheagenic, with a clinical incidence rate of 98% (Peters et al., 2019). Treatment of proliferative organoids with afatinib resulted in marked decrease in cell viability even at low concentrations, resulting in an IC_50_ of 0.036 µM (Figure 2E). The differentiated organoids exhibited far less viability decrease in response to afatinib treatment (IC_50_ of 4.16 µM). With an over 100-fold increase in IC_50_, differentiated organoids are substantially less susceptible to afatinib-mediated toxicity (Figure 2E).

Nifedipine, a calcium channel blocker (Curtis and Scholfield, 2001), is not associated with GIT and was used as an example of a non-diarrheagenic molecule (Peters et al., 2019; Belair et al., 2020). As expected, nifedipine did not affect organoid viability throughout the concentrations tested in either organoid model (Figure 2F).

For all compounds tested, overall toxicity response was consistent between donors. All donors exhibited lower IC_50_ values in the proliferative model than in the differentiated model for afatinib and colchicine, comparable IC_50_ values in the proliferative and differentiated models for sorafenib, and higher IC_50_ values in the proliferative model than the differentiated model for aspirin. Even in cases where there was some donor variability in IC_50_ values, for example, afatinib (Supplementary Figures S2A, B), these trends were conserved.

### 3.3 Organoid differentiation state influences safety margin for diarrheagenicity prediction

**FIGURE 3:**
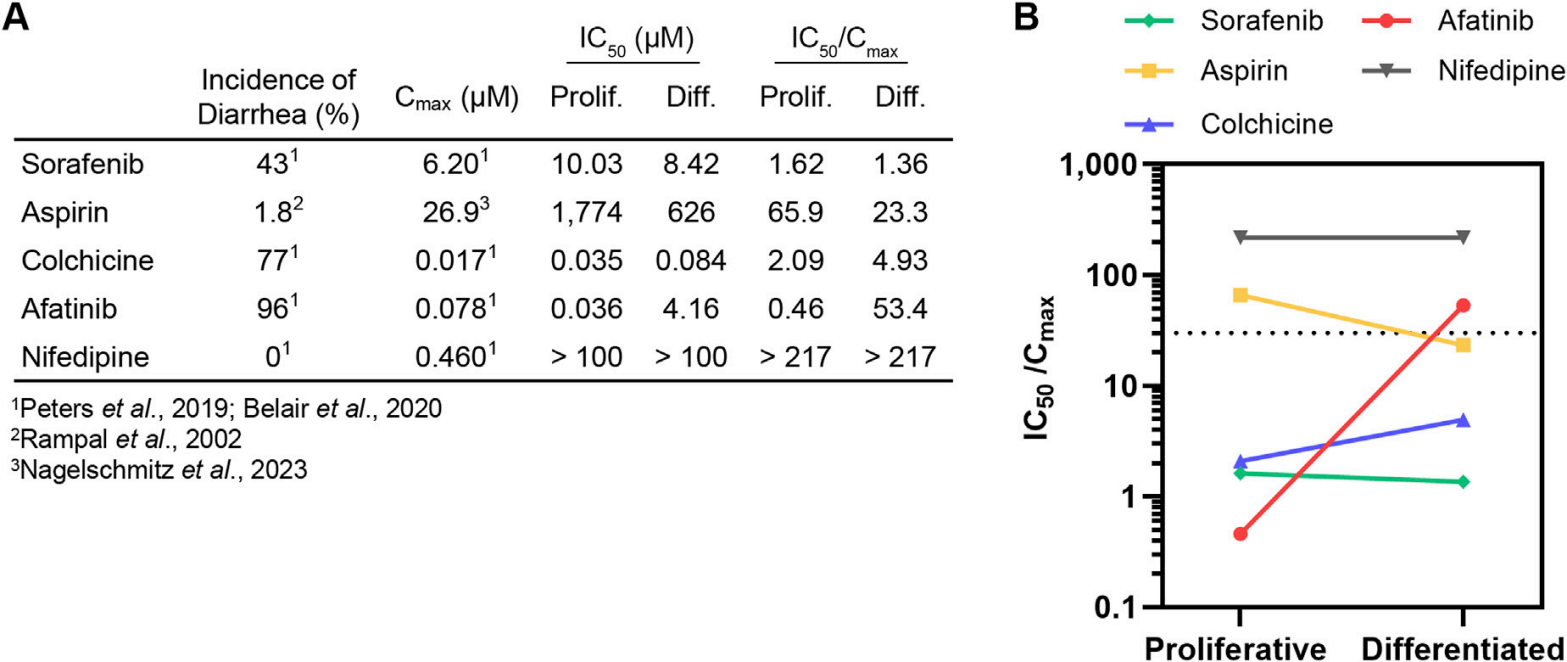
Differentiation state of organoid model can affect diarrheagenicity prediction. **(A)** Summary of clinical incidence of diarrhea, C_max_, IC_50_ (determined in this study), and IC_50_/C_max_. **(B)**. IC_50_/C_max_ in proliferative organoids or differentiated organoids plotted with dotted line that represents IC_50_/C_max_ = 30 safety margin.

While the IC_50_ values generated here (summarized in Figure 3A) provide a comparator for proliferative and differentiated duodenum organoid dose response to a given drug, this metric alone is insufficient to implicate clinical toxicity. To translate *in vitro* toxicity findings to clinical outcomes, previous studies have demonstrated that a drug’s measured IC_50_ divided by its reported maximum plasma concentration (C_max_) in the clinic can be used as a safety margin to predict drug toxicity in the intestine (Peters et al., 2019; Belair et al., 2020) and other organ systems (Redfern et al., 2003; Keating et al., 2014; Proctor et al., 2017; Lehmann et al., 2018; Ridder et al., 2020). In small intestinal organoids, Belair *et al* demonstrated that compounds exhibiting a safety margin lower than 30X the C_max_ could be classified as diarrheagenic (more than 40% incidence of clinical diarrhea) with 90% accuracy (Belair et al., 2020).

To relate our findings to clinical outcomes, we calculated the IC_50_/C_max_ value and categorized a drug as diarrheagenic if the IC_50_/C_max_ value was below 30, the previously established safety margin threshold for small intestinal organoids grown in OGM growth media (Belair et al., 2020). No IC_50_/C_max_ threshold has been previously established to classify compound diarrheagenicity with organoids grown in ODM differentiation media. C_max_ values for all compounds were obtained from previous intestinal organoid *in vitro* model qualification studies (Peters et al., 2019; 2020; Belair et al., 2020) except for aspirin, where the C_max_ value for high-dose oral administration was obtained from a study conducted by Nagelschmitz et al. (2014) (Figures 3A, B). Nifedipine, which had an IC_50_/C_max_ above 30 was correctly classified as non-diarrheagenic in both proliferative and differentiated organoids. Sorafenib was accordingly classified as diarrheagenic in both proliferative and differentiated organoids, as was colchicine despite the higher IC_50_ in differentiated organoids. Aspirin was correctly classified as non-diarrheagenic in the proliferative organoid model, but in the differentiated model aspirin was categorized as diarrheagenic (IC_50_/C_max_ of 65.9 and 23.3, respectively). Afatinib was correctly predicted as diarrheagenic in the proliferative organoid model, with an IC_50_/C_max_ of 0.46. Interestingly, the drastic shift in IC_50_ for afatinib in differentiated organoids resulted in an IC_50_/C_max_ of 53.4, which was above the safety margin threshold of 30, thus incorrectly classifying afatinib as non-diarrheagenic by this metric. Considering that afatinib causes diarrhea in about 96% patients, this is a particularly noteworthy result.

## 4 Discussion

The recent surge in CIVM development unlocks new potential for the pharmaceutical industry, providing physiologically-relevant tissue models to replace transformed cell lines and animal studies to evaluate drug safety and efficacy. As these models are developed further, we must consider how alterations to cell culture conditions affect assay outcomes and interpretability. Intestinal organoids have begun to be adopted to evaluate drug toxicity (Belair et al., 2020), and culture protocols can be optimized to influence the abundance of the major cell types of the intestine (Sato et al., 2009; 2011; Lau et al., 2012; Yin et al., 2014; Gerbe et al., 2016; Fujii et al., 2018; Boonekamp et al., 2020; He et al., 2022; Oost et al., 2023). Because different cell populations may have different toxicity responses to compounds, it is critical that our models represent these cell types and distinguish such MoTs. This may be particularly important for oncology compounds, which are known to target actively dividing cells that a differentiated intestinal model may not capture. Therefore, we developed proliferative and differentiated organoid models of the small intestine to assess how differentiation state of organoids can alter dose response to small molecules in our toxicity assays. In this proof of concept study, we identified three molecules for which toxicity differs in proliferative and differentiation intestine models, highlighting this as an important consideration when choosing models of toxicity.

Primary intestinal organoid and organoid-derived models have proliferative and differentiated cell types, but to varying degrees depending on culture conditions (Fujii et al., 2018; Boonekamp et al., 2020; Oost et al., 2023), tissue segment (Criss et al., 2021; Bieber et al., 2022; Kourula et al., 2023) and donor background (Criss et al., 2021). Model-omics, or high dimensional datasets (such as transcriptomics and proteomics), are important resources to characterize models and contextualize differences in media formulations, culture conditions, passage number, donor tissue and sample site variation for toxicology readouts (Homan, 2023; Kang et al., 2024). We performed bulk RNA-sequencing of the proliferative and differentiated organoid models, and verified that markers of cell proliferation were downregulated upon organoid differentiation, concurrent with upregulation of mature enterocyte and goblet cell markers (Figure 1). Certain cell populations such as enteroendocrine and tuft cells are rare (<1%) in the small intestine *in vivo* (Boonekamp et al., 2020). Additional factors such as cytokines or small molecule notch inhibitors are necessary to enrich for these populations in organoid cultures (Boonekamp et al., 2020; He et al., 2022), but have not been qualified for this assay.

Upon comparison of small molecule-induced toxicity in our proliferative and differentiated organoid models, we found that the differentiation state of the organoid model influenced the IC_50_. In the case of aspirin, the IC_50_ decreased in the differentiated organoid model (Figure 2C). This suggests that differentiated cell types are more prone to aspirin-associated injury than proliferative cells. While the mechanism of epithelial injury is unclear, aspirin is known to interact with enterocytes and damage intestinal villi (Endo et al., 2015). Stem and precursor cells may be more resilient to NSAID toxicity in this case. In contrast, the IC_50_ values of both colchicine and afatinib was higher in differentiated organoids relative to proliferative organoids (Figures 2D, E, respectively). Corroborating this data, a similar finding was noted in another study using a primary tissue-derived monolayer model, in which afatinib and colchicine prevented barrier formation and decreased numbers of proliferating epithelial cells but had little effect on differentiated epithelium (Pike et al., 2024). Despite the difference in platforms and assays, both models demonstrate that proliferative cell populations are more prone to toxicity in response to these compounds, which may be associated with a MoT affecting actively dividing cells, highlighting the importance of cell composition in in vitro models. While the monolayer model provides certain advantages for measuring barrier integrity, organoids remain a more accessible and high-throughput system.

In order to use *in vitro* assays for translational purposes, it is critical to qualify new CIVMs for their intended context of use in toxicity assays against a panel of compounds with known clinical outcomes. A seminal study in this field conducted by Belair *et al* qualified an organoid-based model for predicting GIT, concluding that compounds exhibiting a safety margin (IC_50_/C_max_) less than 30 could be classified as diarrheagenic (Belair et al., 2020). Interestingly, Belair *et al* reported using a differentiated organoid model based on the presence of differentiated cell types by immunofluorescence, however, the IC_50_ values obtained were similar with those we obtained in our proliferative model. It is likely that their model still contained a substantial population of proliferative cells since the organoids were cultured in Intesticult OGM media, which supports cell proliferation, for the entirety of the experiments. In the current study, differentiation was achieved using a commercial media formulation developed to promote cell differentiation. Under these conditions we observed a more differentiated phenotype compared to the proliferative condition by transcriptomics and organoid morphology (Figure 1). IC_50_ values shifted for several compounds tested relative to the proliferative model. This was particularly apparent for afatinib, a highly diarrheagenic small molecule that demonstrated considerably greater toxicity in our proliferative organoid model compared to differentiated organoids (Figure 2E). This difference was strong enough that, using the safety margin cutoff of 30, afatinib would be classified as non-diarrheagenic in the differentiated organoid model (Figures 3A, B). Conversely, aspirin was correctly categorized as nondiarrheagenic using the proliferative organoid model, but in the differentiated model the IC_50_ was lower, resulting in an IC_50_/C_max_ below 30, thus categorizing it as diarrheagenic (Figures 3A, B). This demonstrates two critical points. First, organoid culture conditions and the resulting cell-type compositions can have considerable effects on the assay outcomes and the resulting conclusions. Thoughtful characterization of models is important to understand how to interpret data obtained from them. Second, we need to exercise caution in how we use predictive algorithms to interpret corresponding data. As shown here, a safety margin cutoff for the intestinal organoid model may not be transferable upon changes to culture conditions. Iterations to an existing model intended for predictive toxicology studies warrants new qualification studies to re-calibrate safety margin thresholds to categorize a compound as diarrheagenic.

Another key distinction between the organoid model in the study conducted by Belair *et al* and that presented here is the use of proximal (duodenum) as opposed to distal (ileum) small intestine. Segment-specific differences at the transcriptomic level in tissue are conserved in primary tissue-derived organoids (Middendorp et al., 2014; Zietek et al., 2015), many of which are involved in drug metabolism within the small intestine. Therefore, it would not be surprising if this contributed to regional vulnerability to small molecules. For instance, the more proliferative small intestine has increased sensitivity to oncology compounds in murine organoid-derived monolayers compared to colon-derived monolayers (Bieber et al., 2022). Lastly, we also cannot exclude the possibility that donor-specific differences in any primary tissue-derived intestinal model will react differently to chemical compounds. While dose response curves were similar across the donors tested, we did observe some differences, for example, with afatinib. Differentiated organoids from all donors were less sensitive to afatinib toxicity than their proliferative counterparts, however, the degree of the shift in dose response curves and IC_50_ varied between donors (Supplementary Figure S2).

Several caveats still exist for the 3D primary tissue-derived organoid model. Time in culture is a limiting factor due to spontaneous differentiation of proliferative/stem cells and the finite lifespan of terminally-differentiated cells. Therefore, only acute exposure can be addressed. In addition, only the basolateral surface is exposed to the medium and compounds of interest, while the apical surface is enclosed within the organoid interior. This is a limitation for orally-delivered drugs that would primarily be absorbed at the apical surface. Lastly, epithelial cytotoxicity is only one of several mechanisms of drug-induced diarrhea, and this assay is not suitable to model osmotic or immune-mediated diarrhea (Stein et al., 2010; Keely and Barrett, 2022). Organoid- and primary tissue-derived monolayer models provide apical and basal epithelial access and enable kinetic measurements of barrier integrity (Wang et al., 2017; Peters et al., 2019; Kourula et al., 2023), some of which enable prolonged, regenerative culture conditions (Wang et al., 2022). Furthermore, engineered complex models are being developed to mimic the spatial organization of proliferative and differentiated cell compartments (Nikolaev et al., 2020; Gjorevski et al., 2022; Mitrofanova et al., 2024). Models of even higher complexity that incorporate non-epithelial cell types (such as immune cells and fibroblasts) or biomechanics may be necessary to capture disparate mechanisms of toxicity (Kasendra et al., 2018; 2020; Lin et al., 2023; Recaldin et al., 2023; Kang et al., 2024).

Another consideration for the field is the determination of a safety margin threshold value for predicting drug-induced intestinal toxicity. Currently, total plasma C_max_ is the standard metric used for normalization of IC_50_, but the C_max_ will vary for any given drug based on dose and route of administration. Luminal drug concentration is likely much higher and more relevant than C_max_, especially for orally-delivered compounds, but these values are difficult to obtain (Tanaka et al., 2015; Belair et al., 2020). As observed here and by others (Ridder et al., 2020), this safety margin cannot be generalized to every intestinal CIVM. It will be important to benchmark any of these models against panels of well-characterized compounds with known clinical toxicity outcomes, such as that published by members of the International Consortium for Innovation and Quality in Pharmaceutical Development Microphysiological Systems (IQ-MPS) Affiliate, to develop accurate predictive models for intestinal safety assessment (Peters et al., 2020).

In conclusion, we have demonstrated that the differentiation status of the organoids used for intestinal toxicity assays can meaningfully impact the conclusions drawn, and should be considered when selecting a model and when interpreting safety margin thresholds for predicting toxicity. Using distinct proliferative and differentiated organoid models enables investigation of toxicity responses by both proliferative and differentiated cell compartments, and enables examination of MoTs. More studies will be required to qualify this model and its context of use assay by comparing toxicity in proliferative and differentiated organoids across a larger panel of compounds. When developed intentionally, selected thoughtfully and characterized thoroughly, GI organoids have the potential to improve predictive safety and MoT investigation to advance forward and reverse translation efforts in drug development.

## Data Availability

The datasets presented in this study are available in the National Center for Biotechnology Information Gene Expression Omnibus (NCBI GEO) repository, accession number GSE277613.
