# Supplementary material for: The differentiation state of small intestinal organoid models influences prediction of drug-induced toxicity
Source: Front Cell Dev Biol. 2025 Jan 23;13:1508820. doi: 10.3389/fcell.2025.1508820 (PMC11799252; doi:10.3389/fcell.2025.1508820)
Supplement: Supplementary file 1 [file DataSheet1.docx]

|  | Cell line ID | Tissue | Age | Sex | Reported race |
| --- | --- | --- | --- | --- | --- |
| Donor 1 | GIB002 | Duodenum | 39 | M | White |
| Donor 2 | GIB005 | Duodenum | 55 | M | Black/African American |
| Donor 3 | GIB006 | Duodenum | 60 | F | White |

**Table S1.** **Demographics of donors used in this study**


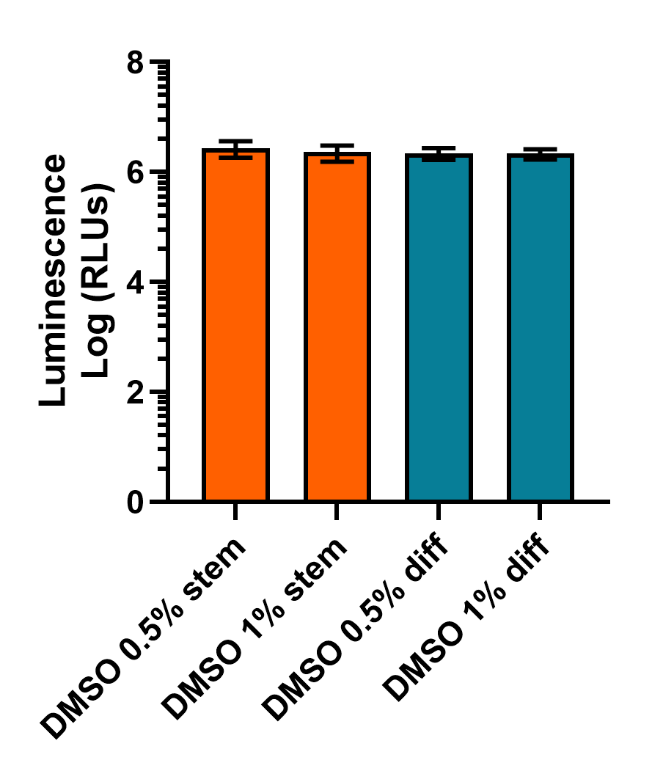


**Figure S1. Proliferative and differentiated organoids exhibit similar viability in response to vehicle controls.** Proliferative and differentiated organoids from 3 different donors were treated with 0.5 or 1% DMSO in corresponding growth or differentiation medium. Relative Luminescence Units (RLUs) were measured using CellTiter Glo (Promega) and averaged across 3 technical replicates. Data represents mean ± SD across at least 2 experiments per donor.


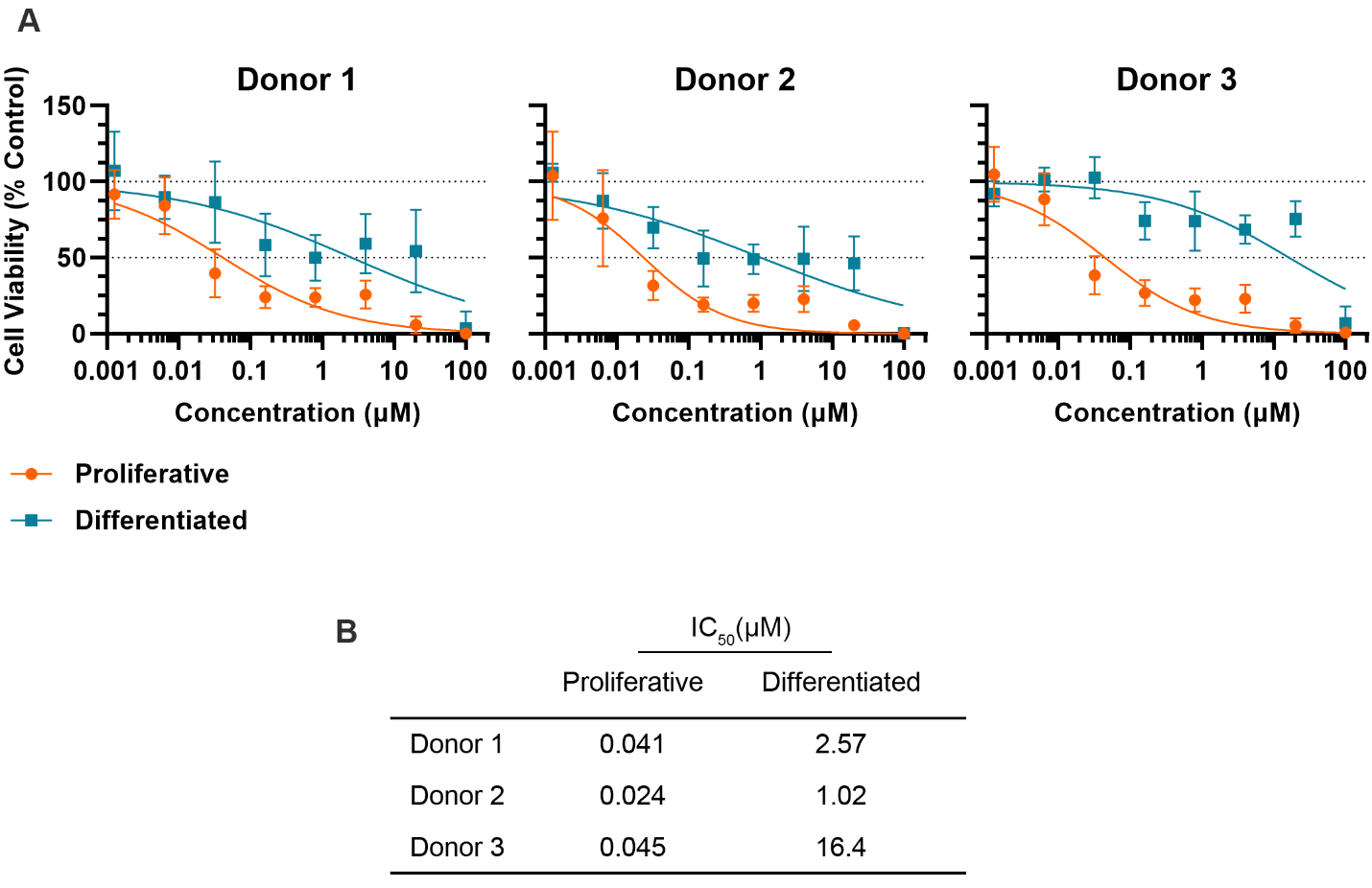


**Figure S2. Donor-specific organoid response to afatinib. (A)** Dose response to afatinib normalized to respective DMSO vehicle control after 3 days of exposure to indicated concentrations of drug. Data represented as mean ± SD of cell viability using CellTiter Glo (Promega) in 3 different donors n>3 replicates. IC_50_ values for afatinib in proliferative and differentiated organoids models summarized in **(B)**.
